# The impact of oxygen supply and erythrocytes during normothermic kidney perfusion

**DOI:** 10.1038/s41598-023-29031-y

**Published:** 2023-02-03

**Authors:** Charlotte von Horn, Hristo Zlatev, Bastian Lüer, Laura Malkus, Saskia Ting, Thomas Minor

**Affiliations:** 1 Department of Surgical Research, University Hospital Essen, Essen, Germany; 2 Clinic for General, Visceral and Transplantation Surgery, University Hospital Essen, Essen, Germany; 3 Department of Pathology, University Hospital Essen, Essen, Germany

**Keywords:** Kidney, Translational research, Kidney

## Abstract

The influence of erythrocytes and oxygen concentration on kidneys during long-term normothermic kidney perfusion is under debate. This study compares acellular and erythrocyte-based NMP with focus on oxygen delivery to the tissue as well as the effects of high oxygenation on tissue integrity. Pig kidneys were connected to NMP for six hours. The first group (n = 6; AC500) was perfused without addition of oxygen carriers, arterial perfusate pO2 was maintained at 500 mmHg. In the second group (n = 6; RBC500) washed erythrocytes were added to the perfusate at pO2 of 500 mmHg. Third group (n = 6; RBC200) was perfused with erythrocyte containing perfusate at more physiological pO2 of 200 mmHg. Addition of RBC did not relevantly increase oxygen consumption of the kidneys during perfusion. Likewise, there were no differences in kidney functional and injury parameters between AC500 and RBC500 group. Expression of erythropoietin as indicator of tissue hypoxia was comparable in all three groups. Cell free NMP at supraphysiological oxygen partial pressure seems to be a safe alternative to erythrocyte based perfusion without adverse effect on kidney integrity and provides a less cumbersome application of NMP in clinical practice.

## Introduction

The steadily increasing shortage of donor organs for transplantation inspires ongoing research for new preservation technologies, particularly for marginal donor organs^1^.

Normothermic machine perfusion (NMP) represents an encouraging tool for isolated organ preservation and reconditioning, also giving opportunities to manipulate grafts prior to transplantation^2–4^. Perfusion at near physiologic conditions can contribute to reduction of hypothermia induced injury, to maintenance of tissue metabolism and potentially enables functional evaluation for prediction of expected graft function after transplantation^4^.

The choice of an appropriate perfusion solution plays an important role during NMP, when organ is metabolic fully active and requires sufficient nutritive support and oxygen supply for energy demanding processes.

The use of whole blood perfusates or erythrocyte supplements during NMP is favored by most research groups and is propagated to be a requisite for efficient oxygen delivery to the graft tissue^3,5,6^. One argument is that in contrast to perfusions with red blood cell based solutions, cell free organ perfusion would need higher flow rates in order to ensure sufficient oxygen provision to tissue, which should be associated with degenerative processes in the perfused tissue^7^. Moreover, it is assumed that red blood cells might exert a colloidal function during long-term normothermic perfusion and promote reduction of edema formation in renal tissue^8^.

On the other hand, blood based perfusion solutions bear different risks and side effects and implicate a more complex application of ex vivo normothermic renal perfusion. Cell contaminations, like leukocytes or inflammatory factors, derived from the blood preparations, may mediate pro inflammatory reactions during reperfusion with adverse effects on graft quality and function^9^. Furthermore, mechanical stress in an artificial system can lead to hemolysis of erythrocytes and release of free hemoglobin, which is known to negatively affect kidney function^5,10^.

Previous studies have elegantly demonstrated that at least short-term perfusions of the kidney can be satisfactorily performed without the addition of oxygen carriers. Using a microfiber optic oxygen sensor it could be shown that tissue pO_2_ at the inner renal cortex remained above normal levels during the whole duration of cell free perfusion provided that the perfusate was equilibrated with 95% oxygen^11^. Moreover, the addition of red blood cells to the perfusate had no beneficial impact on kidney function and innate immune activation compared to the acellular perfusion.

Nonetheless, some researchers have hypothesized that keeping oxygen partial pressure within physiological limits would favor vascular integrity and might be more opportune for isolated organ perfusion than supraphysiological concentrations of oxygen^12,13^. By contrast, others did voluntarily and successfully use oxygenation even of blood based perfusates with supraphysiological oxygen concentrations for ex vivo kidney perfusion^14,15^.

Despite this controversial practice, literature is lacking of methodologically sound investigations taking account of the mutual interrelations of perfusate content in erythrocytes, viscosity, oxygen partial pressure and effective perfusate flow during normothermic kidney perfusion. The present study was undertaken in order to systematically investigate the role of erythrocyte supplements during extended normothermic kidney perfusion with high perfusate oxygenation in both groups. By using a pressure-controlled model, an automatic adaptation of flowrates to the different viscosities of the red blood cell containing and non-containing perfusate should be enabled. A third group with erythrocyte containing perfusate using oxygen partial pressures reduced to physiological levels shall allow for an isolated appreciation of the putative adverse effects of high oxygen concentrations during longer perfusion.

The two questions, scil. if higher flow rate, as occurring upon pressure controlled perfusion with media of lower viscosity, or if supraphysiological oxygen partial pressures would be detrimental to the renal tissue, could thus be addressed in an interleaved approach.

## Materials and methods

The study is reported in accordance to the recommendations in the ARRIVE guidelines (PLoS Bio 8(6), e1000412,2010). All experiments were performed in accordance with the federal law regarding the protection of animals. The principles of laboratory animal care (NIH publication no. 85-23, revised 1985) were followed. The procedure of euthanasia for organ retrieval according to § 4 Abs. 3 TSG (German Legislation on animal protection) has been discussed with the animal welfare officer of the University hospital Essen and has been approved by Landesamt für Natur, Umwelt und Verbraucherschutz (LANUV), NRW, Germany.

In deep anesthesia (ketamine-xylazine), German Landrace pigs weighing between 25 and 30 kg were euthanatized by intra-cardiac injection of KCl. Thereafter, the abdomen was exposed by midline incision and surgical preparation of the kidneys begun. Twenty min after cardio-circulatory standstill, the renal artery was cannulated and the kidneys flushed by 100 cm gravity with 100 ml of HTK-solution on the back-table at 4 °C.

Kidney grafts were randomly assigned to one of the following groups (n = 6, resp.):

1. Direct connection to a pulsatile normothermic machine perfusion for 6 h with Aqix RS I solution supplemented with 40 g/L of bovine serum albumin, 25 mL sodium bicarbonate 8,4% and 4 mg dexamethasone. Perfusion was performed pressure controlled at a mean arterial pressure of 75 mmHg. Amino acid solution (aminomel nephro) was constantly infused by a rate of 1 mL/h. Perfusate was oxygenated with 1L/min of 95% oxygen and 5% carbon dioxide by means of a hollow fiber oxygenator placed in the arterial line of the circuit. Arterial perfusate pO2 was maintained at supra-physiological level at about 500 mmHg. (AC500, n = 6)
2. Direct connection to a pulsatile normothermic machine perfusion for 6 h identical to group 1. 5000 IU of heparin and washed, leukocyte depleted autologous erythrocytes were added to the perfusate to reach a final hemoglobin value of approx. 5 g/dL. As required to maintain renal flow after addition of the red blood cells, Verapamil was infused at a rate of 0.25 ml/h^15^. Perfusate was oxygenated with 1L/min of 95% oxygen and 5% carbon dioxide. Arterial perfusate pO2 was maintained at supra-physiological level at about 500 mmHg. (RBC500, n = 6)
3. Direct connection to a pulsatile normothermic machine perfusion for 6 h identical to group 1. 5000 IU of heparin and washed, leukocyte depleted autologous erythrocytes were added to the perfusate to reach a final hemoglobin value of approx. 5 g/dL. As required to maintain renal flow after addition of the red blood cells, Verapamil was infused at a rate of 0.25 ml/h^15^. Oxygenation of perfusate was regulated to yield a more physiological arterial pO2 of about 200 mmHg^16^. (RBC200, n = 6)

Within each analytical 60 min interval, the produced urine was collected. From every 100 ml of urine, an aliquot of 1 ml was kept for late analysis and the rest was reinfused after filtration (13 µm) at 100 ml intervals to the reservoir in order to prevent alterations in the composition of the perfusate over time, which would be encountered when replacing urine loss by adding balanced salt solution^17,18^.

After every 60 min the last collection of urine was reinfused after 1% of the total volume had been retrieved. The accumulated aliquots were pooled stoichiometrically for determination of creatinine and sodium for later calculation of interval specific clearance or reabsorption rates.

Kidney perfusion pressure was set at 75 mmHg and automatically maintained by a servo-controlled roller-pump, connected to a pressure sensor placed in the inflow line immediately prior to the renal artery.

### Analytical procedures

Analytical routines were performed as described previously^11^. Concentrations of creatinine were determined in a routine fashion by reflectance photometry on a Reflotron Plus point of care unit (Roche Diagnostics, Mannheim, Germany).

Clearances were calculated for the respective intervals as urinary creatinine × urine flow/perfusate creatinine.

The albumin concentration in urine was measured in a routine fashion at the Laboratory center of the University Hospital and the amount of albumin normalized against the corresponding concentrations of creatinine as urinary albumin to creatinine ratio (UACR).

Oxygen partial pressure and perfusate concentrations of sodium and glucose were measured in a pH-blood gas analyser (ABL 815flex acid–base laboratory, Radiometer, Copenhagen).

Renal oxygen consumption [mL 100 g/min] was calculated from arterial and venous oxygen saturations, total hemoglobin and oxygen partial pressures as detailed previously^19^, taking into account the respective flow rates and kidney mass.

The efficiency of renal O_2_ utilization was approximated by the ratio of total tubular transport of Na (TNa) − accounting for the vast majority of energy consuming processes in the kidney^20^-, and VO_2_, with TNa being equal to filtered Na minus excreted Na:

$${\text{TNa}} = \left( {{\text{GFR}} \times {\text{Perfusate}}\;{\text{Na}}} \right) - ({\text{urinary}}\;{\text{Na}} \times {\text{urine}}\;{\text{flow}}).$$

Fractional excretion of sodium (FE Na) has been calculated according to:

$$ {\text{FE}}\;{\text{Na}} = {\text{Na}}_{{({\text{urine}})}} \times {\text{Creatinine}}_{{({\text{perfusate}})}} /{\text{Na}}_{{({\text{perfusate}})}} \times {\text{Creatinine}}_{{({\text{urine}})}} \times {1}00. $$

ELISA kits from Cloud-Clone Corp. (CCC, USA) were used to determine concentrations of high mobility group box-1 (HMGB1) and from LS-Bio (Seattle, USA) for tenascin-C (TNC) for tissue inhibitor of metalloproteinase 2 (TIMP2) according to the instructions of the manufacturers on a fluorescence micro plate reader (Tecan, Grailsheim, Germany).

Oxygen free radical-induced tissue injury was approximated by the amount of thiobarbituric acid-reactive substances (TBARS) in kidney tissue. TBARS were evaluated by fluorimetry from deproteinized samples using the adduct formation with thiobarbituric acid as detailed elsewhere^21^.

### Gene expression analyses

Total RNA was isolated from snap frozen samples and analysed as described previously^22^. The amount of specific mRNA in the tissue was normalized for the respective individual quantities of transcripts of GAPDH, which was analysed as house-keeping gene. Results are expressed as relative deviation from baseline levels that were analysed from native cortical kidney samples processed in parallel. All reagents and primers for GAPDH (n°PPS00192A, Gene ID 396823, Ref. Seq. No. NM_001206359) and EPO (n°PPS00577A, Gene ID 397249, Ref. Seq. No. NM_214134) were purchased from Qiagen GmbH (Hilden, Germany).

### Histology

Kidney tissue was collected at the conclusion of the experiments, cut into small blocks (3 mm thickness) and fixed by immersion in 4% buffered formalin. The blocks were embedded in paraffin, and 2–4 μm tissue slides were prepared using a microtome (HM 325, Microm, Walldorf, Germany). Hematoxylin and eosin (H&E) and periodic acid-Schiff (PAS) staining were conducted adherent to in-house standards and used to assess morphological integrity of the parenchyma.

Assessment was carried out in a blinded fashion in 10 randomly chosen, nonoverlapping fields (× 400 magnification), using a 3-point scale for tubular dilatation, necrosis, edema, inflammation and fluid in tubules: 0 = no damage; 1 = lesions affecting > 25% of the field; 2 ≥ 50%; 3 ≥ 75%. In each individual kidney, the mean value from the 5 respective parameters was calculated and taken as individual mean histological injury score.

### Statistics

All values were expressed as means ± SD. After proving the assumption of normality, differences between the groups were tested by one way ANOVA and post hoc testing with the Tukey Kramer test (Instat 3.01; Graph Pad software Inc, San Diego, CA), unless otherwise indicated. Statistical significance was set at P < 0.05.

## Results

**Figure 1:**
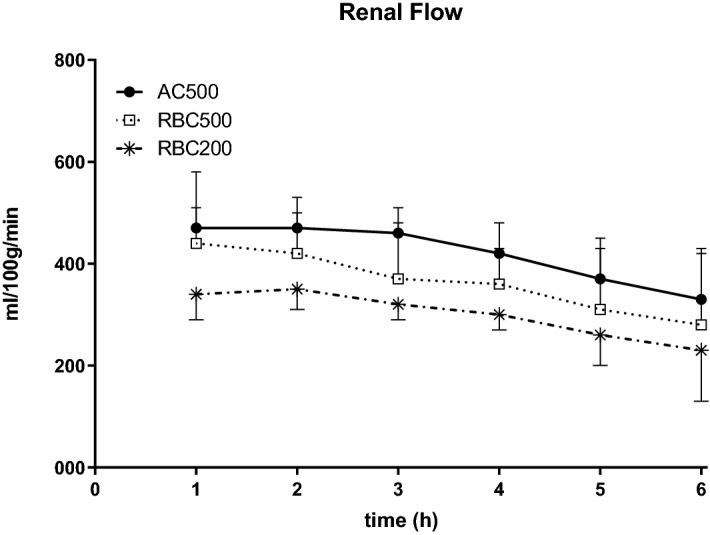
Renal flow values during 6 h normothermic kidney perfusion. Kidneys were perfused with AQIX RS-I without the addition of erythrocytes at pO2 of 500 mmHg (AC500; n = 6), with erythrocyte supplementation at pO2 of 500 mmHg (RBC500; n = 6) and with erythrocyte supplementation at pO2 of 200 mmHg (RBC200; n = 6).

Renal perfusate flow over time in the different groups is depicted in Fig. 1.

No significant differences were observed between the groups. However, highest flow rates have redundantly been seen during the acellular perfusions although vasodilative treatment was only given in the RBC-supplemented groups.

**Figure 2:**
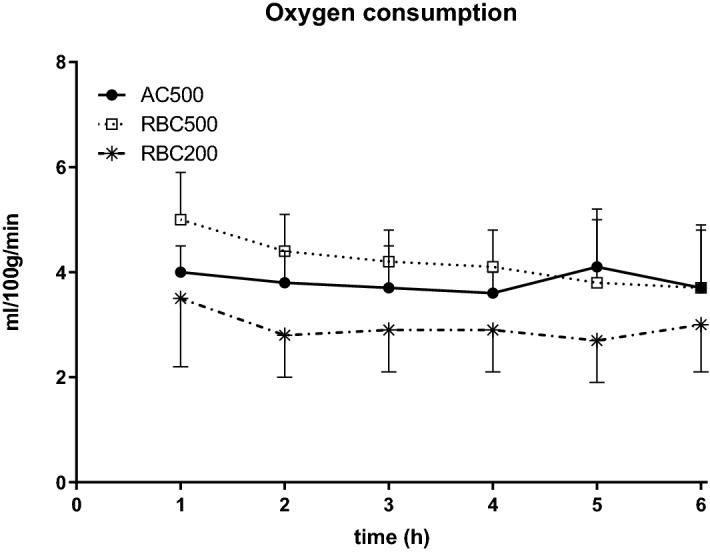
Renal oxygen consumption in the course of 6 h normothermic kidney perfusion. Kidneys were perfused with cell free AQIX RS-I solution at 500 mmHg pO2 (AC500; n = 6) or with erythrocyte containing AQIX RS-I solution at pO2 of 500 mmHg (RBC500; n = 6) and at pO2 of 200 mmHg (RBC200; n = 6).

Oxygen consumption of the kidneys remained stable over time in all groups averaging 3.3 ± 0.9 vs 2.8 ± 1.5 vs 2.3 ± 1.0 µmol/g/min in groups AC500, RBC500 and RBC200, resp. (cf. Fig. 2).

Sufficient oxygen delivery to the grafts throughout the experiments was confirmed by an average venous oxygen partial pressure of 233 ± 69 mmHg in the AC500group, and a venous oxygen saturation of 91 ± 8% in the RBC200 group while oxygen saturation consistently remained at 100% in the RBC500 group.

**Figure 3:**
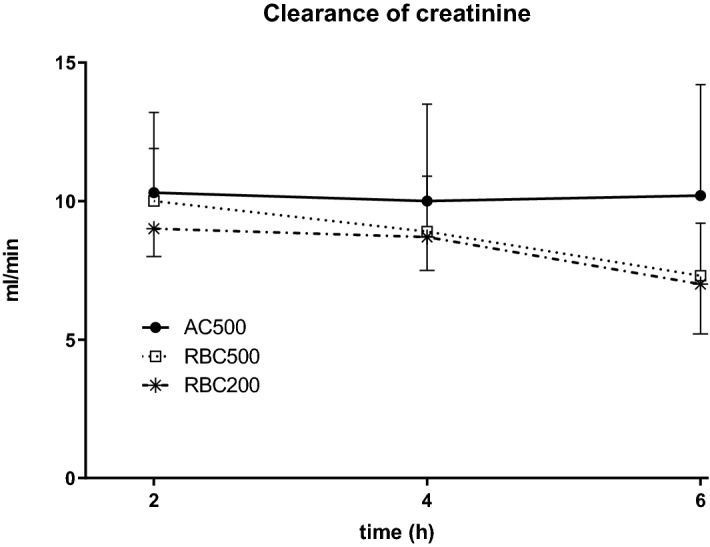
Clearance of creatinine in the course of 6 h normothermic kidney perfusion with AQIX RS-I solution. Perfusion was performed at pO2 of 500 mmHg with (RBC500; n = 6) or without addition of erythrocytes (AC500; n = 6) and with erythrocyte supplemented perfusate at pO2 of 200 mmHg (RBC200; n = 6).

Glomerular function was intermittently monitored by measurement of renal clearance of creatinine during the experiment (cf. Fig. 3). In all groups, clearance of creatinine averaged about 10 ml/min after 2 h of machine perfusion. During the later course of the experiment a slight but nonsignificant decline was observed in the RBC containing groups which was not seen in the AC500 group.

The urine production did not differ significantly between the groups and averaged 4.8 ± 2.1, 5.0 ± 2.0 and 5.1 ± 1.4 ml/min in the groups AC500, RBC500 and RBC200 respectively.

Albuminuria did not differ significantly among the three groups either. Urinary albumin to creatinine ratios at the end of the experiments were 0.08 ± 0.02 vs 0.25 ± 0.25 vs 0.15 ± 0.19 in the groups AC500, RBC500 and RBC200, resp. (p = 0.43 ANOVA).

Preservation of tubular cell function was quite similar during normothermic machine perfusion in all of the three groups (cf. Table 1). Tubular reabsorption of sodium and glucose was sensibly unaffected by the used perfusate. In the same way, the oxygen utilization efficiency i.e. the total amount of transported sodium in relation to the utilized oxygen did not differ between the groups.

**Table 1:** Readouts of tubular cell integrity after 6 h of normothermic machine perfusion with different perfusate composition.

	AC500	RBC500	RBC200	ANOVA
Kidney weight (g)	68 ± 5	64 ± 4	70 ± 8	n.s
FE Na (%)	31.9 ± 15.2	24.9 ± 17.4	33.3 ± 14.6	n.s
FE Glu (%)	38.2 ± 19.3	40.7 ± 15.8	43.0 ± 27.8	n.s
TNa/VO_2_ (µmol/µmol)	8.6 ± 1.3	7.1 ± 1.6	7.0 ± 2.5	n.s
TIMP2 (ng/mL)	1.8 ± 0.5	1.8 ± 0.9	1.6 ± 0.7	n.s

*FE Na, FE Glu* fractional excretion of sodium or glucose; *TNa/VO2* aerobic efficiency-total sodium reabsorption to oxygen consumption ratio, *TIMP2* tissue inhibitor of metalloproteinase 2.

Eventually, no differences between the groups were seen with respect to the leakage of tissue inhibitor of metalloproteinase 2 (TIMP2), recently proposed as early biomarker of renal epithelial injury^23^.

As high partial oxygen pressures in the perfusate might foster oxygen free radical mediated tissue injury we have measured lipid peroxidation (LPO) in the tissue after machine perfusion in the respective groups. Quantification of lipid peroxidation yielded on average 0.87 ± 0.24 nmol/mg vs 0.97 ± 0.27 nmol/mg vs 1.2 ± 0.37 nmol/mg in the groups AC500, RBC500 and RBC200, resp..

On the other hand, perfusion without oxygen carriers could bear the risk of limited oxygen delivery to the tissue, possibly resulting in hypoxic challenges. We therefore looked at the induction of erythropoietin, the mRNA of which is sensibly upregulated in face of tissue hypoxia.

Of note, no significant differences could be substantiated with respect to the expression of erythropoietin after 6 h of machine perfusion with relative values of 0.12 ± 0.1 after acellular perfusion compared to 0.14 ± 0.05 and 0.19 ± 0.09 in the groups RBC500 and RBC200, respectively.

Renal release of danger associated patterns (DAMPs) and thus innate immune activation was followed by the detection of high mobility group box-1 (HMGB1) in the perfusate. Concentrations of HMGB1 tended to be lowest after acellular perfusion (24.8 ± 31.6 pg/ml) compared to 50.9 ± 11.7 pg/ml and 54.6 ± 21.7 pg/ml in the RBC500 and RBC200 groups, resp. but the differences did not quite reach statistical significance.

Moreover, perfusate levels of tenascin C amounted to 0.3 ± 0.2 ng/ml in the AC500 group and 1.0 ± 0.6 ng/ml in the RBC500 group but rose significantly to 2.3 ± 1.1 ng/ml (p < 0.05 vs. AC500 and RBC500) in the RBC200 group. Thus, under the conditions of our study, the reduction of perfusate pO2 to physiologic levels was accompanied by an increased expression of the extracellular biomarker.

Results of the histological examination are outlined in Table 2. Only mild alterations could be seen in all of the kidneys with no significant differences among the three groups. Besides sporadic tubular dilatation or cellular edema some foci of mild to moderate inflammation were documented throughout the three groups.

**Table 2:** Histological injury evaluation by light microscopy at the end of the experiments.

	Tubular dilatation	Necrosis	Edema	Inflammation	Fluid in tubules	Total
AC500	0	0	0	0.5 ± 0.5	0	0.5 ± 0.5
RBC500	0.3 ± 0.5	0	0	0.8 ± 0.9	0.5 ± 0.8	1.6 ± 1.0
RBC200	0.3 ± 0.5	0	0.3 ± 0.4	0.5 ± 0.8	0.3 ± 0.7	1.4 ± 1.7

*AC 500* kidneys that undergo 6 h NMP without oxygen carrier and 500 mmHg oxygen, *RBC 500* kidneys that undergo 6 h NMP with RBC and 500 mmHg oxygen; *RBC 200* kidneys that undergo 6 h NMP with RBC and 200 mmHg oxygen; n = 6 per group.

## Discussion

The supplementation of perfusates with red blood cells or artificial oxygen carriers has generally been considered as a prerequisite for successful normothermic machine perfusion of clinical organ grafts. However, renal perfusions with artificial buffer free of red blood cells have a long standing history in physiologic research and are recognized as suitable model^17,24^ provided that colloidal support and higher oxygen partial pressures are provided.

In our model, we could not substantiate any relevant draw-back in using a cell free colloidal perfusate instead of an RBC supplemented one. If the perfusate was oxygenated to supraphysiological partial pressures of > 500 mmHg, the addition of RBC did not relevantly increase the oxygen consumption of the kidneys. Venous oxygen saturation of the erythrocytes in the RBC500 group continuously remained at 100% (data not shown) and indicated that the RBC did not actively contribute to the oxygen delivery to the tissue. In line with this, mRNA expression of erythropoietin was virtually identic in the groups underpinning that no oxygen deficit had been sensed in the RBC-free perfused kidneys.

Moreover, there were no differences between AC500 and RBC500 with respect to energy dependent tubular cell function like sodium and glucose reabsorption or the release of TIMP2 as early sensor of cellular stress reactions^23^.

Likewise, glomerular function as evaluated by renal clearance of creatinine or albuminuria did not differ significantly between the groups. In this regard, our results differ from previous data reported by Venema et al.^25^. Using porcine slaughterhouse kidneys subjected to 30 min of warm ischemia and hypothermic machine perfusion for 3 h, they found upon subsequent normothermic machine perfusion that adding RBC to the perfusate resulted in favorable effects on fractional excretion of sodium (FENa) while simultaneously compromising renal clearance of creatinine. But then, overall performance of the kidneys was notably compromised in comparison to our model. Of note, in that study, trans-renal flow during normothermic machine perfusion only averaged between 40 and 60 ml/100 g /min, while it reached about 300 to 400 ml/100 g/min in our experiments, similar to values reported by others^15,26^. Flow values less than 50 ml/100 g/min have often been considered a poor outcome criterion and a sign of increased vascular injury or interstitial edema^26,27^. Thus, oxygen consumption was notably limited by the vascular compromise in the former investigation^25^ and amounted only to one third of the values observed in our experiments. Under limited perfusion conditions it may hence occur that the addition of oxygen carriers may gain an importance that it does not have under other circumstances.

Likewise, preliminary studies from us and others did not decipher any difference in renal oxygen consumption or ATP content after normothermic perfusion with or without the addition of erythrocytes^28^, nor did the supplementation of washed erythrocytes result in any functional improvement^11^. In both cases, however, the renal perfusate was equilibrated with 95% of oxygen and such supraphysiologic levels of oxygen had recently been incriminated to endanger tissue viability by maximizing the generation of oxygen free radicals ensuing in radical mediated cellular injury^29–31^.

From a series of 12 consecutive human liver perfusions, 6 with high oxygen tension and 6 at near physiologic values, Watson et al.^30^ suggested avoidance of hyperoxia during perfusion to be associated with lesser risk for vasoplegia upon transplantation. However, his observation could not disclose, whether high oxygen tension or other concomitant parameters were responsible for the outcome differences. Even so, all of the livers in the high oxygen but only 3 out of 6 in the low oxygen arm were DCD organs.

On the other hand, renal perfusions with high oxygen tension have already been experienced by several clinical groups without the occurrence of vasoplegic draw backs^14,26,32,33^.

Beyond that, hypotonus or ischemia are known triggers of flow redistribution from the cortex to the medullary regions in the kidney^34^. The resulting cortical hypoperfusion and heterogeneous flow distribution in peritubular capillaries^35^, create large functional oxygen diffusion distances that are most effectively overcome if physical oxygen partial pressures are high^36^.

Moreover, in our present experiments, we could not disclose any increase in oxygen free radical mediated tissue injury after perfusion with elevated oxygen tension, considered to be the major mechanistic culprit for putative adverse effects of high oxygen partial pressures^30^.

If not for the transport of oxygen, erythrocytes may as well be useful for rheodynamic homeostasis or as colloidal support upon renal perfusion. Thus, blood free perfusion of the kidney may result in non-physiologic filtration fraction^37^ along with an increase of sodium and water excretion after lowering of the hematocrit during renal perfusion^24^.

However, a notable and lasting normalization of sodium reabsorption rates during cellular renal perfusion has been obtained by supplementation of the perfusate with amino acids^38,39^, and the effect of globulins on the fractional excretion of sodium has been shown to be quantitatively at least equal to that observed after addition of red blood cells^8^.

A theoretical advantage of implementing erythrocytes in renal perfusates lies in the possible countercurrent exchange of oxygen from arterial to venous sites in the outer medulla^36^. While high oxygen partial pressures act as driving force to oxygen diffusion across the vasa recta, such diffusion process are limited by high oxygen affinity of hemoglobin resulting in a safer oxygen delivery to the inner medullar regions^36^.

We cannot conclude from our experiments in which manner or up to which extend the implementation of amino-acids or the lack of RBC would affect topical renal performances, but it is of note that overall functional integrity of the blood free perfused organs did not show any deviations from the groups that were supplemented with RBC.

On the other hand, the use of RBC for organ graft perfusion still poses some problems when implemented into clinical practice. A minor inconvenience may be seen in the increased complexity of the perfusion protocol and the necessity to compete for packed erythrocytes.

More important drawbacks are encountered by the risk of hemolysis in the extracorporeal artificial system^10^, resulting in the appearance of free hemoglobin, which in turn may compromise renal integrity and function during ongoing perfusion^10,40^. Moreover, pro-inflammatory stimuli have been reported to be associated with red blood cell transfusions during cardio-pulmonary bypass^41^ and might also be present during extracorporeal organ perfusion.

Novel synthetic oxygen carriers may come up in the future to serve as a more versatile substitute for RBC^42^, but this has not been addressed in our study.

From the present results, however, no stringent evidence could be found to advocate the implementation of RBC in isolated perfusion of the kidney when renal vascular conductivity remains adequate and supraphysiological oxygen tensions are achieved.

High oxygen partial pressures in turn seem rather favorable during isolated perfusion of the kidney, even in presence of RBC as oxygen carriers.

It thus seems quite arguable to extend on the so far scarce but successful clinical attempts^28,32,33^ to take benefit from the logistic ease and immunological inertness of cell free normothermic kidney perfusion.

## Data Availability

The data that support the findings of this study are available from the corresponding author upon reasonable request.
